# The comparison of pro- and antioxidative parameters in plasma and placental tissues during early phase of placental development in cows

**DOI:** 10.1007/s11033-021-06143-0

**Published:** 2021-01-28

**Authors:** Jacek Wawrzykowski, Monika Jamioł, Wioleta Mojsym, Marta Kankofer

**Affiliations:** Department of Biochemistry, Faculty of Veterinary Medicine, University of Life Science in Lublin, Akademicka street 12, 20-033 Lublin, Poland

**Keywords:** Oxidative stress, Pregnancy, Placenta, Cows

## Abstract

Physiological balance between pro- and antioxidative processes is crucial for placentation and further development of fetus and placenta. Parameters of pro- and antioxidative profile may serve as markers of proper course of pregnancy. The aim of study was to assess whether the balance between pro- and antioxidative parameters during placentation phase in bovine placenta is maintained. Placental and blood samples were collected from healthy, HF, pregnant (2nd-3rd month) cows (*n* = 8) in slaughterhouse and in farm, respectively. Formylokinurenine and bityrosine content were measured spectrofluorimetrically in blood plasma and tissue homogenates while metabolites of lipid peroxidation, total antioxidant capacity, SH groups and activity of antioxidative enzymes (glutathione peroxidase and superoxide dismutase) were determined in examined tissues by spectrophotometry. Western blotting was used to confirm the presence of enzymatic proteins in placenta. Results: Local profile in tissues was more pronounced than general profile in blood plasma. Activities of antioxidative enzymes were significantly (*p* < 0.05) higher in 2nd compared to 3rd month of pregnancy in maternal part of placenta while prooxidant parameters showed opposite relationship. Obtained results showed significant differences when compared to data from non-pregnant animals or time of parturition. Further studies are necessary for elucidation of placentation phase in cows.

## Introduction

The balance between pro- and antioxidative processes is crucial for proper functioning of all cells and tissues. It is maintained by different mechanisms which include enzymatic and non-enzymatic antioxidative defense [1]. Enzymatic component covers, among others, glutathione peroxidase (GPx) and superoxide dismutase (SOD). This diversity is indispensable as free radicals are of different character and properties and may attack different molecules [2]. This diversity also makes it difficult to measure antioxidative potential. There are, however, a few methods which can estimate total antioxidative capacity of blood plasma or other tissue fluids. However, the results should be considered as integrated action of anti- and prooxidants.

The balance between production and neutralization of free radicals is a part of physiological, aerobic metabolism of cells. Small amounts of free radicals can be beneficial while their excess is deleterious and leads to an oxidative stress [3]. In consequence, macromolecules are peroxidatively damaged. Proteins, among others, are target molecules for free radicals. Their peroxidative damage may influence important processes and alter physiological functions of proteins including enzyme and hormone action or properties of structural proteins [4]. As this damage may affect selected aminoacids in a protein chain, one of the methods for its estimation is the determination of bityrosine bridges [5]. Peroxidative damage to lipid molecules leads to fatty acid fragmentation and formation of radicals. This in turn may affect cell membranes and their permeability. The intensity of peroxidative processes can be limited by activity of antioxidative enzymes which are able to scavenge free radicals. The comparison of antioxidative activity with the intensity of peroxidative damage can show the range of imbalance.

Protein metabolism occurs also in placental cells during the ovarian cycle [6] and pregnancy. Relatively more is known about the implantation phase while placentation, due to more advanced pregnancy and problems with sample collection, is less explored. Physiological placentation is crucial for appropriate development of the fetus and the course of pregnancy. In cows, it occurs between 25 and 50 days of pregnancy. Protein networking at this time assures adhesion between cells and the cells-extracellular matrix, as well as signaling for vessel development. Moreover, the balance between production and neutralization of free radicals is an important factor for physiological placentation. Searching for biomarkers of this phase would help establish the physiological course of pregnancy or complications at an early stage. Parameters of oxidative stress could be considered as such biomarkers.

The hypothesis stated that oxidative stress may accompany the placentation phase in bovine placenta.

The aim of the study was to compare antioxidative defense (antioxidative enzymes) with the intensity of peroxidative damage to lipids and proteins in order to define the possible level of imbalance leading to oxidative stress. Moreover, the presence of enzymatic proteins for selected antioxidative enzymes, as well as their molecular weight in placental tissues, were detected.

## Material and methods

### Placental tissues and plasma samples

Placental tissues were collected in local slaughterhouse from healthy HF cows (*n* = 8) in 2–3 month of pregnancy. Cows were of 4–6 years old and veterinary examined. The crown-crump length of the fetus served for the estimation of pregnancy age. Pregnant uteri were removed and transported immediately to laboratory. Uteri were opened always at the same location and 3 placentomes close to cut were selected and removed.

Parturient tissue samples (*n* = 5), serving as controls, were collected from healthy animals subjected to routine elective caesarian section. Care was taken to cut samples always from similar location in uterus, as well as similar fragments of placentome. Maternal and fetal parts were separated manually.

Blood samples were collected during routine veterinary inspections from healthy HF cows (*n* = 8) in 2nd-3rd month of pregnancy. Non-pregnant cows (n = 8) served as controls. Blood samples were collected into tubes with anticoagulant (EDTA) via puncture of the jugular vein and centrifuged. Obtained plasma samples were portioned and stored at −20 °C until analyzed.

The information about the presence of pregnant uterus occurred postmortem and it was impossible to collect blood sample. That is why blood samples were collected at local farm.

All institutional and national guidelines for the care and use of animals were followed (EU Directive 2010/63/EU for animal experiments). Experiments on tissues collected in slaughterhouse postmortem do not require approval of ethical commission. Collection of biological material during routine veterinary procedures does not require approval of ethical commission.

### Enzyme determinations

Individual samples were homogenized in Ultra Turrax (Ikawerk, Janke, Kunkel, Staufen, Germany) on ice with PBS (0.1 mol/dm^3^, pH = 7.0), Triton X-100 and protease inhibitor cocktail (Sigma, Poland). Homogenates were centrifuged at 4 °C and 6500 x g for 20 min. Obtained supernatants were portioned and stored at −20 °C for subsequent Western blotting as well as enzyme activities.

#### GPx activity determination [7]

Incubation mixture contained: 2.58 ml phosphate buffer (0.05 mol/dm^3^, pH = 7.0), 100 μl 8,4 mol/dm^3^ NADPH (Merck), 10 μl glutathione reductase (100 U/mg protein, Sigma), 10 μl 1.125 mol/dm^3^ sodium azide (Sigma) and 100 μl 0.02 mol/dm^3^ glutathione (Sigma), 100 μl of homogenate as well as 100 μl 0.022 mol/dm^3^ H_2_O_2_ as substrate. The change of absorbance of NADPH which was converted to NADP^+^, was measured between 2nd and 4th minute of reaction at 340 nm (Ultrospec 2000, Pharmacia, Sweden). The calculation was based on standard curve prepared with different concentrations of NADPH. Enzyme activity was expressed in nanokatals (nkat) per protein content.

#### SOD activity determination [8]

Quartz cuvette contained 1.8 ml carbonate buffer (0.05 mol/dm^3^, pH = 10.2), 100 μl homogenate and 100 μl adrenaline as substrate (18 mg/10 ml 0.1 mol/dm^3^ HCl, Sigma). The method was based on the inhibition of the spontaneous degradation of adrenaline to adrenochrom at pH 10.2 by SOD. The increase of absorbance measured at 340 nm (Ultrospec 2000, Pharmacia, Sweden) during 10 min incubation was compared with control where homogenate was replaced by 0.9% NaCl. Enzyme activity was expressed in SOD units (U) per protein content.

#### TAC determination [9]

Working reagent was prepared immediately before use and consisted of 300 mmol/dm^3^ acetate buffer (pH = 3.6), 10 mmol/dm^3^ 2,4,6-tri-pyridyl-s-triazine (TPTZ, Sigma, Poznan, Poland) in 40 mmol/dm^3^ HCl and 20 mmol/dm^3^ FeCl_3_ x 6H_2_O mixed in the ratio of 10:1:1.

Working reagent (2250 μl) was mixed with 25 μl of supernatant or plasma and absorbance was measured at 593 nm (Ultrospec 2000, Pharmacia, Sweden) against the working reagent alone. After exactly 10 min of incubation at room temperature, the absorbance was read again. The difference in absorbance at zero and 10 min time was compared with standard curve prepared with different dilutions of Fe (II) – 0 – 1000 μmol/dm^3^. The analysis of TAC was based on ferric reducing ability of sample. The changes in absorbance were directly related to the “total” reducing power of the electron donating antioxidants present in examined samples. The results were recalculated per protein content of supernatants and expressed as μmol/g protein.

### The determination of products of peroxidative damage to proteins and lipids

#### The determination of DEPPD radicals [10]

The method was based on the estimation of radical cation formed in the reaction of alkoxy and peroxy radicals derived from the hydroperoxides by use of N,N-diethyl-para-phenylene diamine (DEPPD, Sigma, Poznan, Poland). Incubation mixture contained 1 ml acetate buffer (pH = 4.8), 10 μl aqueous solution of DEPPD (0.37 mol/dm^3^) and 20 μl supernatant or plasma. After 1.5 h incubation at 37 °C absorbance was read at 505 nm (Ultrospec 2000, Pharmacia, Sweden) against distilled water. Control sample contained distilled water instead of supernatant. Calculations were based on standard curve prepared with different dilutions of H_2_O_2_. The results were expressed as μmol/g protein.

#### The content of sufhydryl groups [11]

A volume of 300 μl 10% (*w*/*v*) sodium dodecyl sulphate (SDS, Sigma, Poznań, Poland) in 10 mmol/l sodium phosphate buffer (pH = 8.0) was added to 300 μl of sample and mixed precisely. A 2.4 ml of 10 mmol/l sodium phosphate buffer (pH = 8.0) was added. Then 300 μl 20 mg of 5,5-dithiobis-2-nitro benzoate (Sigma, Poznań, Poland) in 50 ml of buffer (DTNB) was added and the absorbance was measured at 412 nm (Ultrospec 2000, Pharmacia, Sweden). The control sample contained 300 μl of the same buffer instead of DTNB. All samples were incubated for 1 h at 37 °C. After incubation, the absorbance was measured again at 412 nm. The difference in absorbance before and after incubation (after subtracting the respective absorbance of the control) referred to the content of SH groups. The content was calculated using a standard curve prepared with different dilutions of glutathione (GSH, Sigma, Poznań, Poland) ranging from 0 to 1 mmol/l in 10 mmol/l sodium phosphate buffer (pH = 8.0) and expressed in mmol/g protein.

#### The content of formylokinurenine and bityrosine bridges [11]

Formylokinurenine (FOR) and bityrosine bridges (BIT) were determined by a spectroflurimetric method. The diluted plasma and homogenate samples were excited by light at 360 nm and 325 nm and emission was measured at 454 nm and 410 nm wavelength, respectively for FOR and BIT. The spectrofluorimeter (Jasco, Tokyo, Japan) was standardized to 100 deflections with chinine sulphate (0.1 μg/ml in 0.1 mol/ H_2_SO_4_) at excitation (350 nm) and emission wavelength (445 nm). The results were expressed as μg/mg protein.

### Western blotting

Ten micrograms of pooled protein samples were boiled at 95 °C for 10 min in loading buffer (0.5 M DTT, 10% (*w*/*v*) SDS, 0.4 M Tris-HCl pH = 6.8 and 50% (*v*/v) glycerol). Samples were separated using a 15% (w/v) Bis-Tris) and running in Protean Mini Tetra (Bio-Rad Laboratories, Hercules, CA, USA) at 200 V for 50 min. Gels were blotted on to Trans Blot Turbo nitrocellulose membranes (Bio-Rad Laboratories, Hercules, CA, USA) at 100 V for 60 min in Criterion Blotter (Bio-Rad Laboratories, Hercules, CA, USA). After incubation with Bloxall blocking solution (Vector Laboratories, Burlingame, CA, USA) at 4 °C for 1 h and washing 3 times with Tris-buffered saline containing 0.1% Tween 20 (TBST), primary antibody, 1:200 sheep polyclonal anti-bovine GSH-Px antibody (4690-4004, Anti-bovine GSH-Px (Biogenesis Ltd., Poole, UK) or 1:2000 rabbit polyclonal anti-rat Mn SOD antibody (600-401-G13, Anti-Mn SOD Antibody) was applied at 4 °C overnight. After washing, membranes were incubated with 1:10000 alkaline phosphatase conjugated goat anti-rabbit antibody (ab6722, Abcam, Cambridge, UK) or rabbit anti-sheep antibody (ab6748, Abcam, Cambridge, UK) respectively at 25 °C for 1 h. The expressed proteins were visualized using NBT/BCIP reagents. The immunoblot signals were scanned (GS-710, Calibrated Imaging Densitometer) and analysed by Ouantity One 4.1 software (Bio-Rad Laboratories, Hercules, CA, USA). The western blotting was performed three times.

### Protein determination

Plasma protein content was determined by use of Lowry’s method [12]. The method was based on coloured reaction between aromatic aminoacids and Folin-Ciocalteu reagent.

### Statistical analysis

Statistical analyses were done using non-parametric Kruskal–Wallis [13] and Mann–Whitney [14] U test. For this purpose, the IBM SPSS Statistics v21 software (IBM Corporation, USA) was used. The results were presented as mean ± SD. Significance was declared if *p* < 0.05. Plasma samples taken from pregnant cows were analyzed, non-pregnant animals were used as controls. Placental tissues (maternal and fetal part) were compared separately.

## Results

The results of pro/antioxidative profile in homogenates of placental tissues are given in Table 1. Initially 8 cows were included into one group but statistical analysis allowed for further division into 2nd and 3rd month due to significant differences detected between examined cows.

**Table 1:** Selected pro and antioxidative parameters in maternal and fetal part of bovine placenta in 2nd and 3rd month of pregnancy

Parameter	2nd month Maternal Mean (SD)	3rd month Maternal Mean (SD)	2nd month Fetal Mean (SD)	3rd month Fetal Mean (SD)
GPx nkat/g prot	61.76 (17.38) a	42.51 (15.24) aA	73.44 (27.57)	89.73 (10.46) A
SOD U/g prot	1.26 (0.26) aA	0.75 (0.04) aB	1.90 (0.21) A	2.14 (0.26) B
TAC μmol/g prot	27.22 (3.95)	21.83 (5.21) A	31.50 (6.30)	32.46 (4.11) A
DEPPD mmol/g prot	0.11 (0.01) A	0.14 (0.040) A	0.32 (0.03) aA	0.28 (0.02) aB
SH mmol/g prot	0.024 (0.004)	0.028 (0.003)	0.026 (0.006)	0.032 (0.003)
BIT μg/mg prot	0.229 (0.03) a	0.304 (0.01) aA	0.223 (0.08)	0.245 (0.02) A
FOR μg/mg prot	0.042 (0.010)	0.043 (0.005) A	0.058 (0.007)	0.057 (0.003) A

a, a – significant differences between months within the same part of placenta (GPx maternal - *p* < 0.05; SOD maternal - *p* < 0.001; DEPPD fetal - *p* < 0.05; BIT maternal - *p* < 0.05)

A, A; B,B – significant differences between maternal and fetal part of placenta within the same month (2nd month: SOD - *p* < 0.05; DEPPD - *p* < 0.05; 3rd month: GPx, SOD, TAC, DEPPD - *p* < 0.05; BIT- *p* < 0.05; FOR - *p* < 0.05)

Enzyme activities and TAC values decreased from 2nd to 3rd month in maternal part while slightly increased in fetal part in the same time period. Simultaneously the products of peroxidative damage to proteins and lipids slightly increased in maternal part and reminded similar in fetal part.

Increasing trend in the content of SH groups was observed in examined tissues and different time points. As examined parameters were closely related to each other the results should be considered together and even if changes were not significant their trends were in agreement with significant changes observed in other parameters.

The results of pro/antioxidative profile in blood plasma are shown in Table 2. TAC values were significantly (*p* < 0.001) different between pregnant and not pregnant animals. DEPPD values differed significantly between examined months (*p* < 0.05) as well as between pregnant and non-pregnant cows (*p* < 0.001).

**Table 2:** Selected pro- and antioxidative parameters in plasma of cows in 2nd and 3rd month of pregnancy

month	TAC (μmol/g) Mean (SD)	BIT (μg/mg) Mean (SD)	FOR (μg/mg) Mean (SD)	DEPPD (mmol/g) Mean (SD)
2nd	4.80 (0.82) a	0.082 (0.010)	0.020 (0.003)	0.043 (0.015) a
3rd	4.40 (1.09) a	0.090 (0.017)	0.023 (0.004)	0.030 (0.016) b
NP (non-pregnant)	2.81 (0.34) b	0.092 (0.019)	0.021 (0.004)	0.007 (0.005) c

Different letters mean significant difference between examined time points

**Fig. 1:**
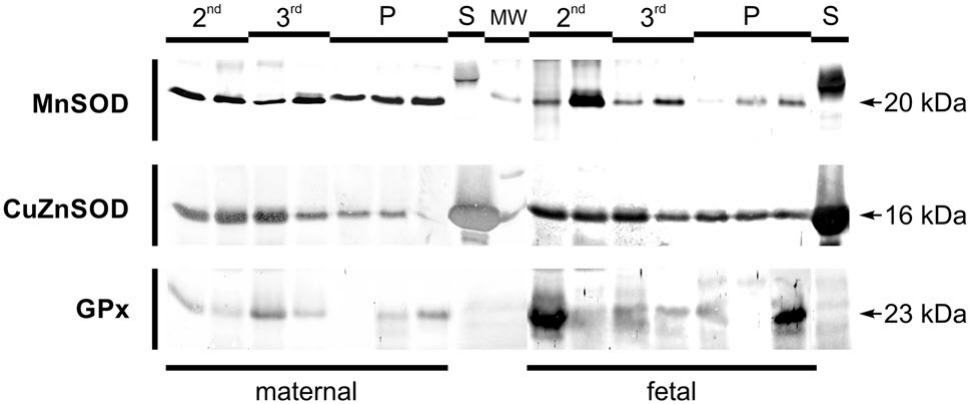
Enzymatic proteins of antioxidative enzymes in bovine placental tissues at 2nd and 3rd month of pregnancy and at parturition (P) One band at 16 kDa for CuZnSOD, one band at 20 kDa for MnSOD and one band at 23 kDa for GPx, S – enzymatic protein standard, MW – molecular weight marker.

Figure 1 provided with the results of Western blotting which confirmed the presence of protein molecules of examined enzymes in placental tissues. As was shown, MnSOD, CuZnSOD and GPx proteins were detected in bovine placenta in 2nd and 3rd month of pregnancy and at parturition (band at 20 kDa, 16 kDa and 23 kDa, respectively), corresponding to the molecular weight of the bovine proteins. Lane P represented samples from parturition and served as the control.

## Discussion

The manuscript, for the first time, described the comparison of anti- and prooxidative parameters in bovine placenta as well as in blood plasma during early-mid pregnancy. The presence of protein molecules of antioxidant enzymes was confirmed in placental tissues during early-mid pregnancy.

Anti- and prooxidative profile of tissues and/or plasma can be used as a biomarker of appropriate functioning of cells and tissues. Moreover, the determination of activity of antioxidative enzymes can be used for the evaluation of oxidative stress [15].

A rise in oxygen tension which may occur during early pregnancy is associated with morphological changes in uterine arteries and the increase in expression (mRNA and activities) of antioxidative enzymes in placental tissues. A burst of oxidative stress is supposed to be related to the establishment of placental circulation [16]. Koç Öztürk et al. [17] provided additional evidence that during pregnancy the increase in tissue oxygen requirements occurs. It might accompany the increase in ROS synthesis and turnover and in turn could lead to the oxidation of SH groups and changes in other examined parameters of protein peroxidation. It is known from human studies that ROS are necessary for the proliferation of trophoblast and its invasion as well as angiogenesis. Moreover, hypoxia may lead to changes in integrin expression and activity of metalloproteinases, which in consequence may cause pregnancy complications related to alterations in adhesion. Studies on pro- and antioxidative balance in plasma in early human pregnancy (10 weeks) revealed that the decrease in antioxidant defense accompanied pregnancy complications [18]. For more details see the review of Wu et al. [19].

The value of results obtained here can be appreciated when compared to other time points e.g. pregnancy or parturition as well as when compared with pro- and antioxidative parts. The results of determinations related to parturition were published previously by our team [20, 21]. Enzyme activities increased close to parturition in both parts of the placenta (GPx - 89.5/134.1 nkat/prot and SOD - 4.4/2.9 U/prot in the maternal and fetal parts, respectively) in comparison to present data. TAC values increased in the maternal part (40.5 μmol/g) while they were similar in the fetal part (28.0 μmol/g) in comparison to present data. Differences between maternal and fetal parts which were previously noticed and at present could be connected with a different histological structure of the placenta as well as different functions in the placental development.

The comparison between two examined months related to the placentation phase in tissues showed the imbalance between anti- and prooxidant balance, because enzyme activities decreased together with the increase in the content of peroxidative damage to macromolecules. Whether it results from the increase in peroxidative processes or decrease in the antioxidative defense requires further elucidation. The placentation phase requires concert action of cellular communication between maternal and fetal components of the placenta for the formation of an adequate environment for the fetus and all these reactions can be part of this process. Any alterations could be related to pregnancy complications associated with disturbed adhesion and invasion. The comparison between placentation time (present data) and parturition (previous data) showed that the imbalance between anti- and prooxidant reactions continued to deepen together with the progress of pregnancy.

Values determined in blood plasma can be compared both with non-pregnant animals and parturition time. A present experiment confirmed significant increase in TAC and lipid peroxidation in plasma during the placentation phase in comparison to non-pregnant animals. It could indicate that antioxidative defense answered to increased peroxidative reactions. Results of previous studies on periparturient cows revealed an increasing tendency in TAC values in plasma in comparison to the 2nd-3rd month of pregnancy [22]. The studies on the comparison of pro- and antioxidative parameters in blood plasma and saliva of pregnant, non-pregnant and sexually immature cows confirmed results obtained here regarding TAC. Some of the observed differences could be related to a different environmental and feeding regime [23].

Animals examined in the present study remained under certain, known hormonal status related to pregnancy, in particular placentation. That is why the evidence that antioxidative enzymes are under the control of steroid hormones may partly explain the differences in their activities [24, 25]. It is known that estrogens themselves exert antioxidant activity, probably by non-genomic stimulation of antioxidative enzymes as well as scavenging action against free radicals [26, 27]. Moreover, materno-fetal auto protection against free radicals was detected by Reyes et al. [28] in in vitro experiments with the use of 17β-estradiol and estriol. The authors explained this protection by the fact that pregnancy is supposed to be associated with oxidative stress.

The content of SH groups can be an important marker of protein peroxidative processes. The decrease in their content within time may reflect protein damage while the increase may indicate the intensification of antioxidative defence against free radicals. SH groups are common for the majority of proteins as they are present in cysteine and are necessary for maintenance of the structure of the molecule. They are susceptible to attack of free radicals and may react with a wide range of them as well as with electrophilic compounds [29]. The present study showed relatively stable concentrations of SH groups in plasma which are probably protected from peroxidative damage by multifunctional enzymatic and non-enzymatic antioxidative systems.

Obtained results indicate that pro- and antioxidative processes are more pronounced locally in tissues than in blood plasma. The differences in profile between non-pregnant, pregnant and parturient cows may indicate that the measurement of oxidative stress parameters in blood can be of value as biomarkers of physiological or complicated pregnancy.

Although this study allowed for the detection of only partial differences in the examined parameters and their sources, which would have a statistically significant level, the tendency of changes in the intensity of pro- and antioxidative processes is interesting in the similarity it bears to oxidative stress. Further studies on full pregnancy time are needed to compare the results between physiological and complicated pregnancy.
